# Type 2 Diabetes, Obesity, and Cancer Share Some Common and Critical Pathways

**DOI:** 10.3389/fonc.2020.600824

**Published:** 2021-01-20

**Authors:** Ishrat Rahman, Md Tanwir Athar, Mozaffarul Islam

**Affiliations:** ^1^Department of Basic Dental Sciences, College of Dentistry, Princess Nourah bint Abdulrahman University, Riyadh, Saudi Arabia; ^2^Scientific Research Center, Prince Sultan Military Medical City, Riyadh, Saudi Arabia

**Keywords:** type 2 diabetes, cancer, shared genes and proteins, breast cancer, ovarian cancer

## Abstract

Diabetes and cancer are among the most frequent and complex diseases. Epidemiological evidence showed that the patients suffering from diabetes are significantly at higher risk for a number of cancer types. There are a number of evidence that support the hypothesis that these diseases are interlinked, and obesity may aggravate the risk(s) of type 2 diabetes and cancer. Multi-level unwanted alterations such as (epi-)genetic alterations, changes at the transcriptional level, and altered signaling pathways (receptor, cytoplasmic, and nuclear level) are the major source which promotes a number of complex diseases and such heterogeneous level of complexities are considered as the major barrier in the development of therapeutic agents. With so many known challenges, it is critical to understand the relationships and the commonly shared causes between type 2 diabetes and cancer, which is difficult to unravel and understand. Furthermore, the real complexity arises from contended corroborations that specific drug(s) (individually or in combination) during the treatment of type 2 diabetes may increase or decrease the cancer risk or affect cancer prognosis. In this review article, we have presented the recent and most updated evidence from the studies where the origin, biological background, the correlation between them have been presented or proved. Furthermore, we have summarized the methodological challenges and tasks that are frequently encountered. We have also outlined the physiological links between type 2 diabetes and cancers. Finally, we have presented and summarized the outline of the hallmarks for both these diseases, diabetes and cancer.

## Introduction

Diabetes and cancer are classified under the most complex diseases. A number of epidemiological studies suggest that diabetic patients are significantly at higher risk for cancer (1–6). The combination of these illnesses is the most challenging in terms of diagnosis because of its heterogeneous and complex nature. In a simplified way, it can also be said that cancer and/or diabetes are due to the failure at multiple levels in multicellular organisms [due to genetic lesions, abnormal signaling, post-translational modifications (PTMs), and metabolic disorders]. These changes are a potential cause of altered cell-fate decisions (proliferation, apoptosis, growth, differentiation). After such changes, the cell appears to be clearly different in physiological behavior and morphology compared to the respective normal cells.

**Figure 1:**
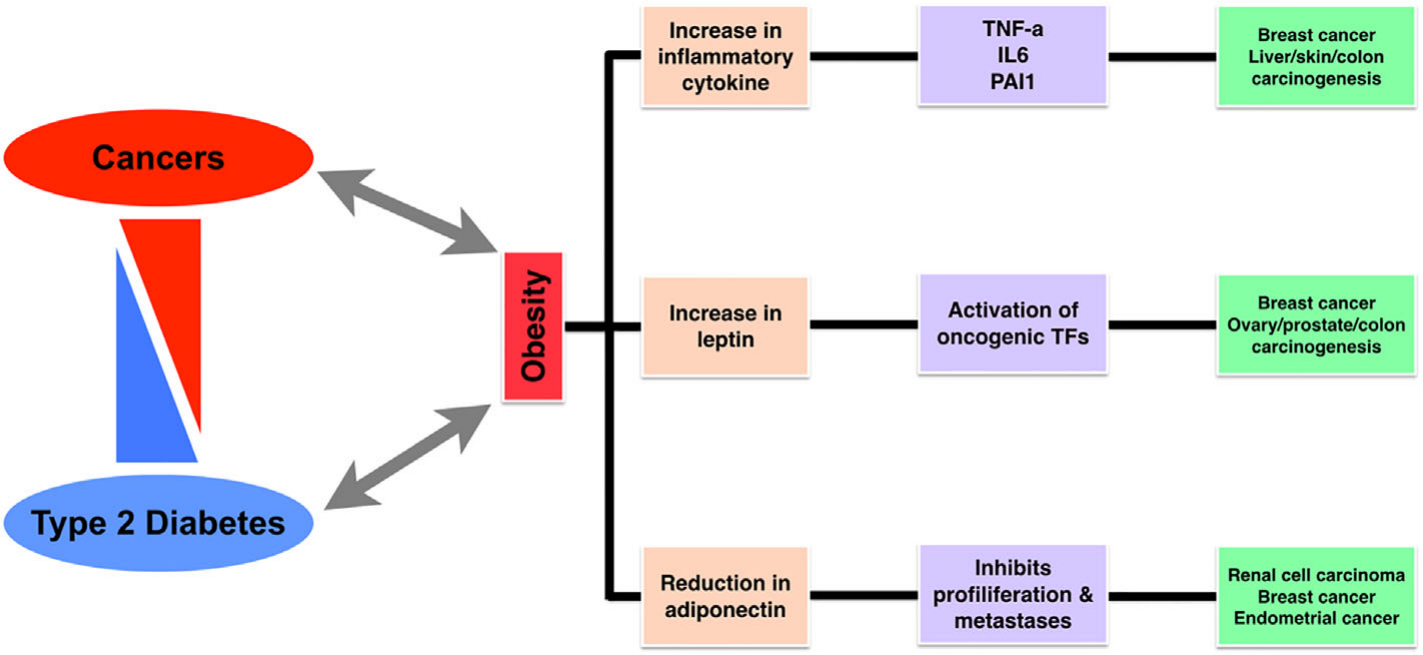
A diagram representing the association between cancer, diabetes, and obesity, followed by shared markers.

The (epi-)genetic alterations (1–8) and transcriptional level change have a potential impact on cellular signaling pathways and networks. In cancer and diabetes, several genes or proteins are mutated, resulting in suppression or overexpression, and consequently undergo conformational changes such as post-translational modifications. It leads to altered cellular signaling pathways and functions, finally leading to the alteration in metabolic processes (1, 3, 9–17). The simultaneity of obesity and type 2 diabetes (T2D) with the growing number of different types of cancer patients from different demographical populations has motivated scientific communities in unraveling the epidemiological, biological evidence and relationships, and diagnostic biomarkers between such complex diseases. Generally, oncologists need to plan a better diagnostic approach for cancer patients who are also suffering from other comorbidities such as diabetes and obesity. Similarly, clinicians also need to adopt a different therapeutic approach for diabetic patients treated and simultaneously suffering from cancer(s). Biologically, T2D and cancers are associated with an abnormality in the PI3K–AKT signaling pathway (mainly at mTOR level), mostly upregulated in neoplastic tissue and downregulated in insulin target tissues in case of T2D. In **Figure 1**, we have graphically illustrated and summarized precisely the association between obesity, diabetes, and cancers. In the majority of the therapeutic approaches, for diabetes and cancers, PI3K pathway functioning is differential where in diabetes treatment its activation is necessary, while in cancer treatments its inhibition is needed. Based on such perspective, we could say that inadvertent emanations of antidiabetic therapy and its effect on tumor cells, or *vice versa*, are presumptive (18).

## Historical and Epidemiological Links

We mentioned earlier the basic conceptual link between obesity, T2D and cancers. There are also historical and epidemiological associations between T2D and cancers. A French surgeon Theodore Tuffier revealed the first initial associations between diabetes and cancer (18, 19), where he studied how diabetes may affect cancer prevalence or the cellular programming of cancer and *vice-versa*. Based on a previous study, a number of facts have been shown which directly relate T2D and different types of cancer. One of the most common points is that it has been established that obesity may promote a state of chronic inflammation and hence, insulin resistance leading to T2D and the chronic inflammation may cause DNA damage and lead to cancer (19–24).

In short, we could summarize from global data about obesity, T2D, and cancer patients that from day-to-day, there is an exponential increase in the incidence of obesity, diabetes, and cancer mainly in the past few decades. These incidences’ trends display the potential evidence of the association between obesity, diabetes, and cancer. In most cases, the risk of cancers and the mortality rate rise collaterally because of the increase in obesity and diabetes rates (3, 25, 26). Such a pattern concludes that the research concerning the biological links between them and the clinical patient management for those who are suffering from both these diseases need to be deeply understood and focused.

## Origin of T2D and Cancer

There are several studies where the origin of T2D and cancers have been discussed individually and together (11, 27–29). People with T2D are almost double as conceivable for liver and pancreatic cancers and also have a higher risk for developing breast, bladder, and colon cancers. There is a higher mortality rate in diabetic women with breast cancer, while diabetic men have a lower risk of developing prostate cancer. Here, we have presented a well-studied phenomenon of the origin of T2D where T2D is mainly characterized by insulin resistance followed by relatively reduced secretion of insulin, and in general, with the little known information about the real mechanism of the abnormal response from the body tissues against insulin involves the insulin receptor (IR). T2D is globally the most common type of diabetes mellitus, and the majority of patients have evidence of “Prediabetes” for many years before meeting the criteria for T2D (30, 31). Prediabetes means impaired fasting glucose and/or impaired glucose tolerance and can be reversed back to normal by a number of precautions, diet control, and selective application of drugs for improved insulin sensitivity and reduced glucose production level (21, 32–34).

**Figure 2:**
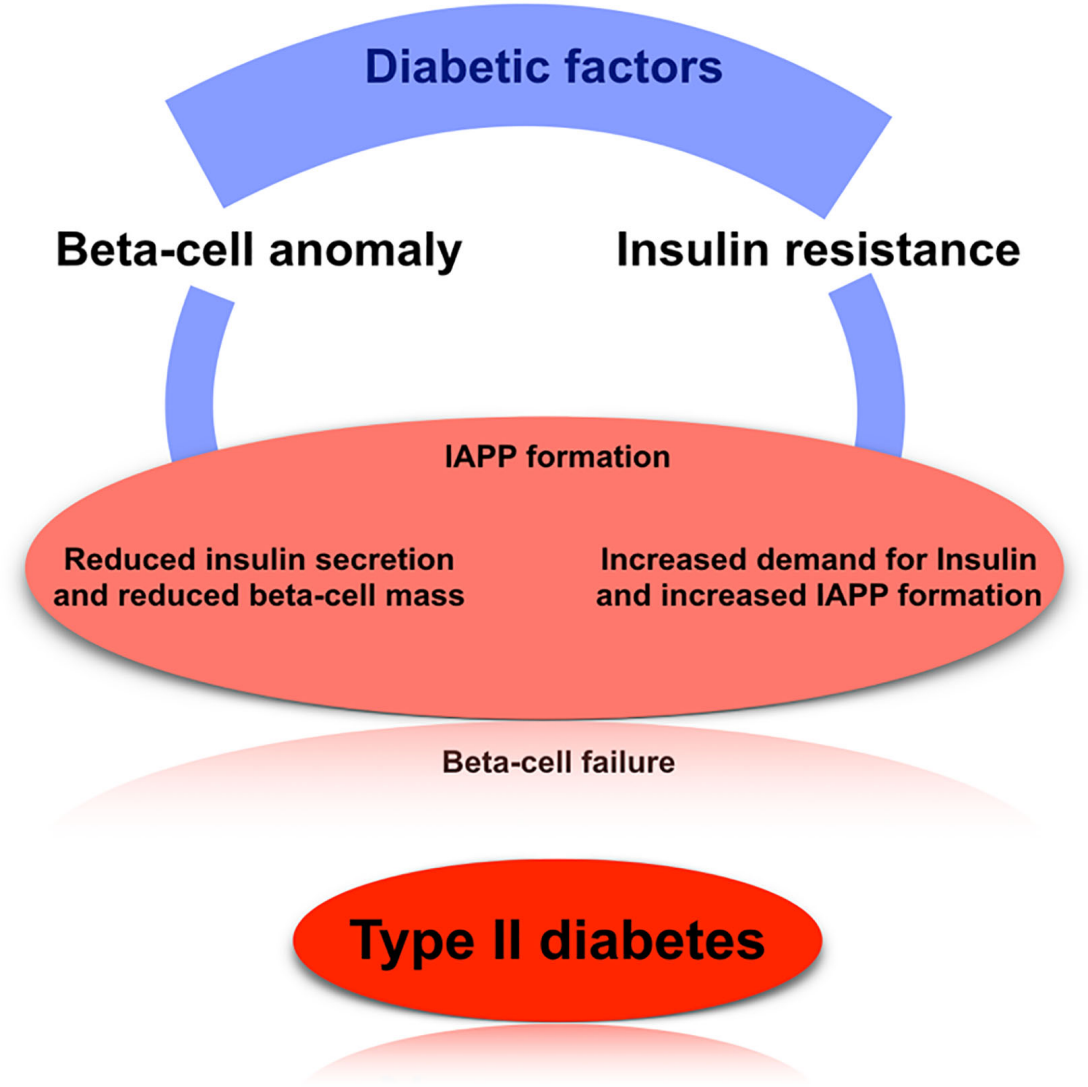
The basic mechanism of type 2 diabetes. Here, we have shown the well-established mechanism of how T2D arise in case of human, which mainly focus on beta-cell anomaly, insulin resistance, and IAPP formation.

T2D factors are mainly associated with lifestyle factors and also the genetic reasons, where the lifestyle factors are obesity, lack of physical activities, poor diet, and stress. The diabetogenic factors (both genetic and environmental) are considered as the potential source of abnormality of the islet beta cells’ function. A defect in the beta-cells leads to reduced insulin sensitivity and increased insulin resistance, causing a higher insulin demand; clinically observed as hyperinsulinemia during the prediabetic stage (19, 35, 36–38). When simultaneous amyloid polypeptide production is adequately sizeable enough and continues for a longer time period, then the formation of the amyloid islet is induced, which may lead to the damage or degeneration of the beta cells. Due to the continuous insulin resistance and reduction of insulin secretion ability, an enhanced response in the normal beta cells occur to increase insulin and islet amyloid peptide levels. If not controlled the cyclic events may lead to increased amyloid level and decrease in insulin production capacity by the pancreas. Insufficient insulin production (in terms of the continuous insulin resistance or failure of beta-cell) leads to an increased concentration of serum glucose. Thus amyloid polypeptide acts as an endogenous diabetogenic factor that leads to the dysfunction of beta-cell and abnormal insulin production (39–42). Controlling T2D may include preventing and controlling the process of amyloid-related beta-cell failure at the early stage, by inhibiting the overproduction of human islet amyloid polypeptide or inhibiting fibrillogenesis (29, 42–46) (**Figure 2**).

Proliferation (cell division) and apoptosis (cell death) are regular and normal processes used by the body for maintaining growth and repair (47), and healthy cells stop cell division when not required (controlled cell division) while in the case of cancer cells there happens to be uncontrolled cell division (proliferation) due to multi-step aberrations, leading to the formation of tumors. This uncontrolled proliferation leads to the metastasis process, which helps in the spreading of cancer from one organ to another (11). The cancer cells could be grouped under different categories based on the cell type and its origin, such as carcinoma (epithelial in origin), leukemia (blood cells), myeloma and lymphoma (originate from the cells of the immune system), sarcoma [originates in connective tissues (bone, muscle, and fat)], mesothelioma (originates in the mesothelium), central nervous system (CNS) (originate from cells body and spinal cord) (1, 11, 48).

## Obesity, Diabetes, and Risk of Cancer

Obesity is one of the most frequent health-associated problems globally, and it is usually quantified by using BMI, although it is not a perfect or highly accurate measure of adiposity. The patient with a higher BMI has increased risks in multiple types of cancers such as pancreatic, ovarian, endometrial, kidney, liver, gallbladder, esophageal adenocarcinoma, and postmenopausal breast cancer. It is also known that obesity is not a key factor in the origin of all the tumor cells (*e.g.*, testicular cancer). Moreover, there are also few cancers that appear to be less common in the case of obesity (lungs, head and neck), and it could be concluded that weight gain may act as one factor associated with cancer progression of different organs. Different from weight gain, the impact of weight loss is not well established about cancer risks (49).

Similarly, T2D diagnosis is linked to increased risk for a set of different cancers, and evidence suggests the associations between pre-diagnostic T2D and incident breast or colorectal cancer. TD2 has also been linked to liver and pancreas cancer incidence rates. Since these cancers are obesity-associated it is possible that common factors could be a possible source of relationships for obesity and diabetes as well as different cancers. The majority of T2D patients are obese and are receiving different therapies for obesity, diabetes, and cancers. The underlying risk is difficult to be evaluated and remains a primary concern for the clinicians (20).

It is also known that diabetes is linked with lowering the risk of prostate cancer. One potential explanation could be that T2D under the treatment of metformin gives favorable outcomes in terms of reducing the chances of prostate cancer. Therefore, it proves the positive role of metformin in reducing prostate cancer (50, 51).

## Biological Mechanisms

**Figure 3:**
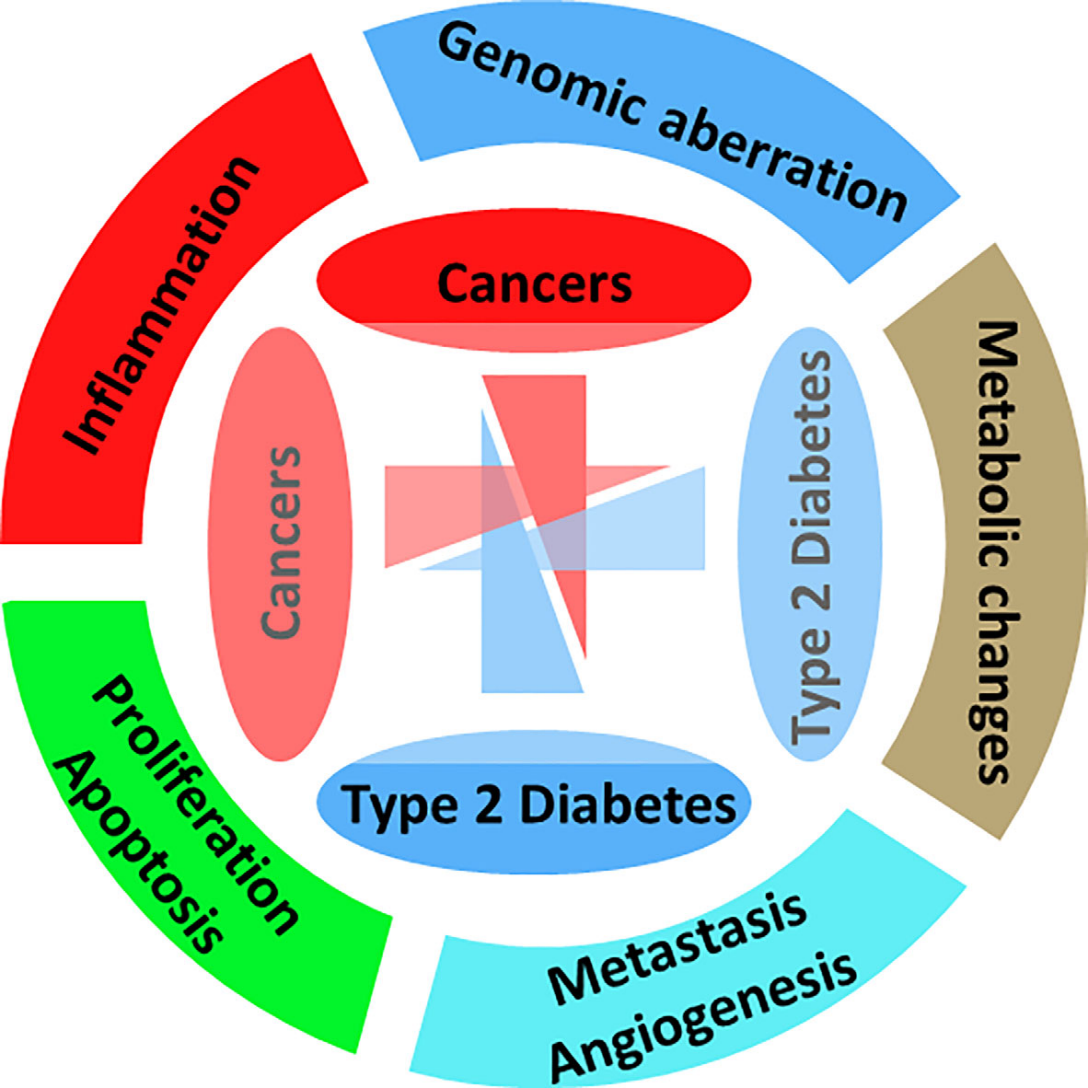
Hallmarks of diabetes and cancer. Representing the major affected biological processes, pathways, and mechanisms.

There are a number of previous studies with detailed current hypotheses and mechanisms with links from obesity and diabetes to neoplasia, as shown in **Figure 1**. One of the most common concepts is that the abnormal endocrine status of obesity and T2D may promote cancer growth, development, and/or its aggressive behavior. **Figure 3** gives an overview of the pathophysiological processes and mechanisms common to both diabetes and cancer. The major pathway components such as insulin, insulin-like growth factors (IGFs), cytokines, and adipokines act as a bridge or the mediators for these three diseases. Additionally, insulin also has an impact on carbohydrate metabolism in different types of tissues (fat, muscle, and liver). It is also known that hormone (mainly peptide) may act as a mitogen in the case of epithelial cells that express IGFR. In the case of T2D and obesity, there are increased insulin levels, and due to increased insulin levels as well as insulin resistance, it is considered as an increased cancer risk (51, 52).

**Figure 4:**
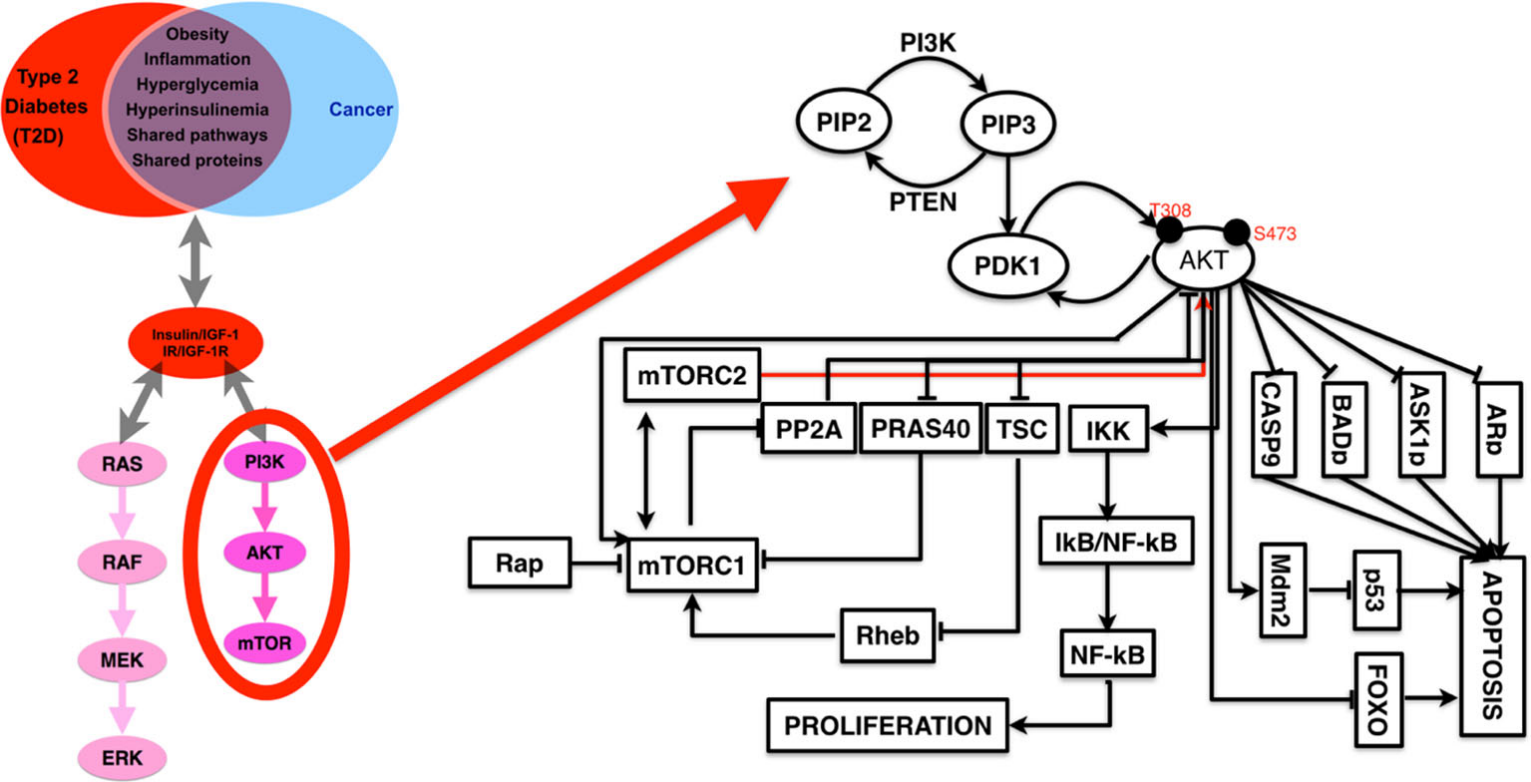
A detailed representation of PI3K–AKT pathways with the list of known interactor partners that appear to be a major link (in terms of common cause and therapeutic targets) between cancer and diabetes.

Based on previous literature, we have presented a summarized figure where we have shown the common and two well-studied signaling pathways for the insulin and insulin receptor and IGF-1 and IGF-1 receptor as well as for both cancer and diabetes (53–57). As in **Figure 4**, we see that PI3K–Akt and MAPK pathways are downstream to IGFR, so we may link the IGFR expression and genetic changes directly to cancer because these pathways are well-studied for cancer. The evidence suggests that PTEN increases PI3K–Akt and MAPK signaling pathway and has increased cancer risk compared to those without this genetic aberration and who are also hypersensitive to insulin and obese. It also suggests that insulin receptor triggering is associated with cancer risk. So, PI3K–Akt signaling is critical for both the cases of carcinogenesis and obesity. In addition, there are also other pathways which are known to be associated with diabetes and cancer both, and one of the highly studied and targeted pathway is RhoA signaling pathway (7, 58, 59).

## Common Links Based on Gene Expression Dataset

**Figure 5:**
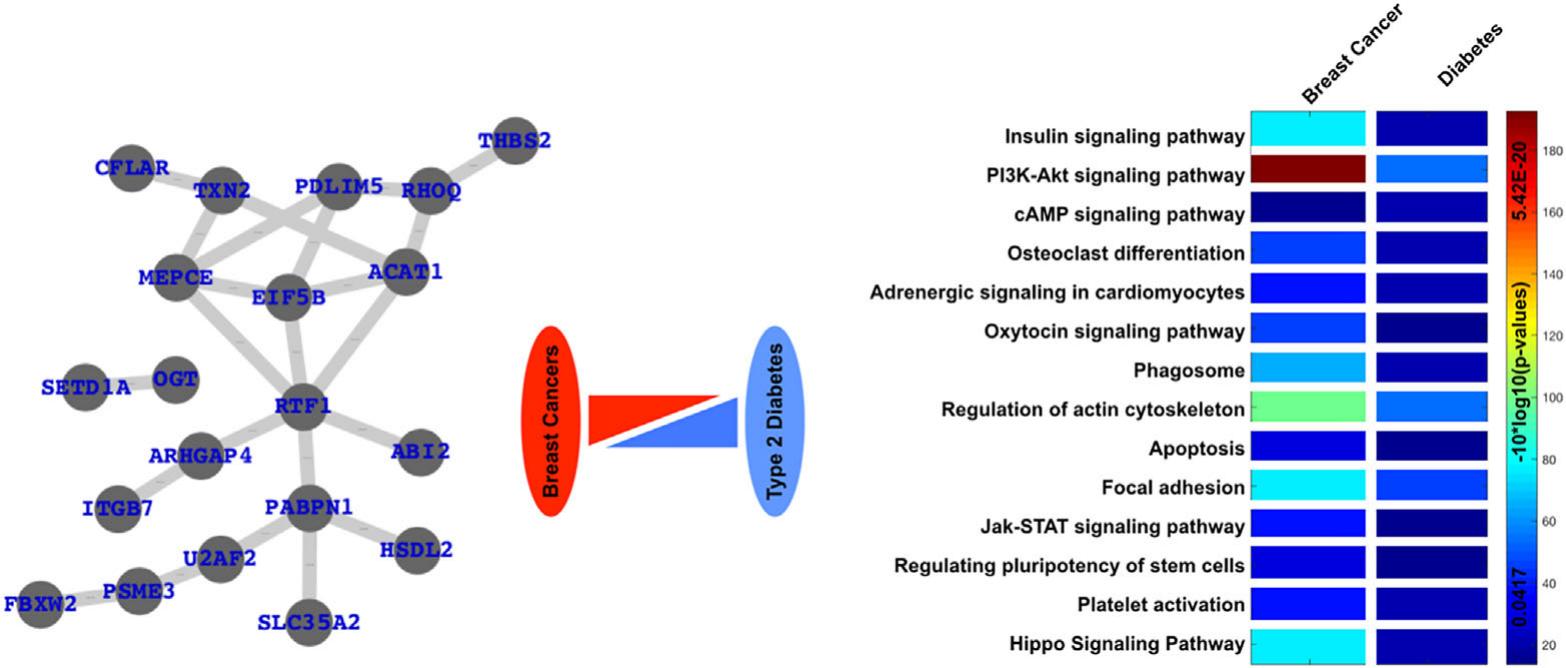
Common links or genes and pathways are based on the analysis of freely available gene expression datasets. Here, we have clearly presented a network of commonly differentially expressed and commonly enriched pathways in case of diabetes as well as one type of cancer, *i.e.*, breast cancer.

Here, we have selected the gene expression datasets available from GEO (gene expression omnibus), one sample from human diabetes and one sample from human breast cancer, analyzed the differentially expressed genes (DEGs) and the enriched pathways for both the datasets and finally, analyzed the common DEGs and enriched pathways (**Figure 5**). Here, we clearly see that these two specific diseases share a number of genes and pathways or are commonly altered in both cases.

## Antidiabetic Therapy and Cancer Risk

In the previous sections, we have introduced three highly complex diseases, obesity, diabetes, and different types of cancers and their association with each other. In addition, we have also presented the summarized biological mechanism. Now we have presented a short brief about the cancer risk during diabetic therapy. There are a large number of drugs used against T2D and are known to be associated with cancer risk. For example, metformin is known to be associated with low cancer risk. Other anti-diabetic drugs such as rosiglitazone are also reported to have anticancer properties in breast cancer cell line (60), however there are other drugs that are associated with higher cancer risk. The drugs known to be associated with high cancer risk are insulin glargine, pioglitazone, sulfonylureas, GLP-1R agonists, and DPP4 inhibitors (61, 62).

## Cancer Treatments, its Effect, and Diabetes Outcomes

In the case of cancer treatment, there are a number of factors that need to be considered before prescribing the drugs, mainly when the patients are suffering from an additional disease such as diabetes. So there is always a possibility that when we proceed with the therapeutic approach, we find that the drug was not entirely therapeutic for either of the diseases, thus it is important to understand the impact of the drugs in such complicated cases. In such cases a different therapeutic approach may be required, such as cancer immunotherapy and herbal therapy, which may be mutually beneficial. Intensive treatment of different types of cancers may be curative, but there is also evidence about the increased risk of diabetes for long-term survivors, even if the underlying mechanism is not clearly known (63–65).

In the case of cancer prognosis, the well-known side effects are cachexia and weight loss, which are considered negative disease outcome factors, while it is also known that a high BMI during diagnosis for different cancers is associated with increased mortality. Pre-diagnostic body mass index (BMI) is associated with various cancer types. The risk factor of BMI varies with the metabolic, hormonal, or immune processes and finally could be the source for neoplastic disease (66). In addition, post-diagnostic weight gain is known to be associated with increased mortality level for the patients who have breast cancer, and also the endometrial cancer rate of mortality is highly dependent on obesity, and additionally, there is evidence which suggests that prostate cancer patients who are in the top quartile in terms of bodyweight have a significantly higher risk of mortality. In summary, we could say that a higher baseline of bodyweight negatively affects the outcome of the cancer treatment.

## Diabetes Prognosis and its Effect

It is established that there are a number of therapeutic approaches for both cancer and diabetes. Many drugs are available for use in diabetes and metformin is among the most frequently used. There is also an increasing trend for the use of natural therapeutic agents. Hence, it is critical to understand the side effect of the application of such treatment option(s).

### Metformin

Metformin is one of the known drugs given to T2D patients. Furthermore, it has also been reported that metformin reduces the circulatory androgens. Consequently, the risk of prostate cancer can also be minimized with this drug. In addition to this, several other cancer types such as HER-2^+^ breast cancer and ovarian cancers have also been modulated by metformin (67–70). The literature-based study also provides the evidence for murine models where it appears useful against the treatment of bladder cancer. The effectiveness of oral metformin is of promising and potential interest, and its use is known for improved recurrence-free survival after radical cystectomy where the patients have diabetes as well as bladder cancer (20, 71).

### Glucocorticoids

Glucocorticoids [a class of corticosteroids (class of steroid hormones)] are among the well-known class of drugs that concern glycemia. Glucocorticoids are used in oncological practice as antineoplastic drugs, to halt the development of certain cancers. It is known to play different roles, which include reduced intracranial pressure in cancer of different subtypes of neurological cancers such as gliomas and meningiomas and also reduces cancer-associated pain (67, 68, 72). Glucocorticoids may lead to insulin resistance as a result of the reduced level of transcription and phosphorylation for the major insulin receptors IRS proteins and because of its downstream position and are considered as the most critical component for signal transduction by this receptor. Steroid-associated hyperglycemia may also be considered a potential source for promoting hepatic gluconeogenesis. The prognostic pertinence of the above-mentioned biological impact is not clearly known, while hyperglycemia is well-known as a poor prognostic factor in the case of infants and children who are mainly suffering from acute lymphoblastic leukemia. Based on these studies, we could consider that tumor cells could manage and satisfy their need for glucose under normal conditions (normoglycemic) and that increased glucose-level (hyperglycemia condition) does not give additional benefit for potential growth advantage (20, 73).

### Hormone Therapy

There is a commonly used therapeutic approach used for prostate cancer treatment known as androgen deprivation/suppression therapy, where gonadotropin-releasing hormone (GnRH) agonists are used. Low levels of testosterone act as one of the factors for higher insulin resistance risk, *i.e.*, hyperinsulinemia and the metabolic disorders (such as diabetes, high blood pressure, and obesity) in men, and testosterone levels are considered to be linked to body mass composition. It has been known that androgen deprivation therapy (ADT) use which is associated with diabetes (mainly the patients suffering from prostate cancer) show poor prognosis to diabetic therapy after ADT of one year (74–77).

### Insulin Signaling Pathway Inhibition

The insulin signaling pathway is an important pathway, and its components are associated with many critical signaling pathways associated with cancers, immune systems, and more. This pathway acts as a source for increasing glucose uptake and reduction of glucose synthesis. The factors which may influence this pathway are fasting, stress, and hormones, and it has been a major focus of understanding because of this pathway’s association with a number of diseases diabetes, hyperglycemia, and hypoglycemia (13, 78–81). It is clearly established that insulin may be the cause behind reduced glucose-level by the activation of the IGF-1R and some of the downstream signaling cascades, mainly PI3K and MAPK pathways in the liver, fat, and muscle, and it was leading to the increased glucose uptake and reduced gluconeogenesis which results in lowered glucose level in blood.

AKT–PI3K pathway is a thoroughly studied pathway in case of cancer, and there are a number of targets from this PI3K–AKT pathway (known to drive neoplastic behavior) for which drugs are available (55, 82–86). It is known that insulin receptors’ family members are considered as the key activators for the PI3K–AKT pathway and are frequently activated by oncogenic episodes (mutations, loss of function, change in gene expression pattern) and few examples are overexpression of HER2, loss of PTEN function, and abnormal mutations in PI3K (2, 78, 87–90). There are a number of drugs and/or inhibitors for targeting the PI3K–AKT pathway and insulin-signaling pathway, and here we have discussed some of them. There are a number of biological macromolecules which may be used for targeting diabetes and cancers such as fucoidan, and their potential targets are known to be associated with diabetes and cancers (91).

### Rapamycin and Rapalogs

The most common mTOR inhibitors are temsirolimus and everolimus used for different types of cancer such as HER2-negative breast cancer, pancreatic neuroendocrine tumors, estrogen-receptor-positive, and renal-cell carcinoma (92–94). The metabolic toxicity mechanism of rapalogs is complex, which includes reduced insulin secretion from the beta cells and also insulin resistance. Since the unraveling of rapalogs’ effect, oncologists are more careful in considering the metabolic toxicity risks. Metformin, which has been discussed above, is considered as the preferred option for the targeting orapalog-induced hyperglycemia (mainly for hyperinsulinemia patients) (92, 95–98).

## Conclusions

Here, we present the relationship between diabetes and cancer in summary as a hallmark of both diabetes and cancers (1). Based on previous studies and reviews, we could see the relationship between obesity, T2D, and cancer is of potential interest and shares the functions and the pathways and particularly the pathway components at a broader scale. It is also clear that maintaining a BMI is important for the prevention and precaution of diabetes, and there is evidence that supports the idea that obesity may act as an increased cancer risk. Previous works also suggest that there is a comparatively higher death risk for cancer patients suffering from diabetes in comparison with non-diabetic patients, and increased cancer mortality rate with diabetes is deeply affected by the cancer prevalence, and because of the shared pathways and pathway components, there is also the possibility of being influenced during the diagnosis, medications, and therapeutic approaches for either of the diseases. It is clearly established that the broad association between both types of patients, *i.e.*, diabetes with cancer or cancer with diabetes prognosis, is critical and is of high priority from a public health point of view. The point which remains unanswered is how does diabetes influence cancer treatment? There are also established facts that obesity induces chronic inflammation and also insulin resistance leading to T2D (19–21), and the risk of different types of cancers has also been linked with obesity and T2D while determining whether diabetes or antidiabetic therapy could act independently from obesity in terms of influencing the risk and also the prognosis of cancers as a potentially challenging task.

In this work, we have summarized the most recent studies regarding obesity, T2D, and cancer relationship and also have presented the summary in graphical forms (**Figure 1**) of the processes, pathways, and components followed by the therapeutic approaches and the risks of drug therapy and specifically known inhibitors. This work will not only help the clinicians but also the researchers to design new experiments and explore more biomarkers as potential drug targets for the development of new inhibitors and drugs for T2D and cancer.

## Author Contributions

IR conducted the literature search and data retrieval. MA analyzed the data, designed the pictorial of the pathways, and participated in the writing of the manuscript. MI analyzed the data and wrote the manuscript. MI and IR critically revised the manuscript and gave the final approval. All authors contributed to the article and approved the submitted version.

## Conflict of Interest

The authors declare that the research was conducted in the absence of any commercial or financial relationships that could be construed as a potential conflict of interest.
